# A conserved and essential basic region mediates tRNA binding to the Elp1 subunit of the Saccharomyces cerevisiae Elongator complex

**DOI:** 10.1111/mmi.12624

**Published:** 2014-05-19

**Authors:** Rachael Di Santo, Susanne Bandau, Michael J R Stark

**Affiliations:** Centre for Gene Regulation & Expression, College of Life Sciences, MSI/WTB Complex, University of DundeeDundee, DD1 5EH, Scotland, UK

## Abstract

Elongator is a conserved, multi-protein complex discovered in *Saccharomyces cerevisiae*, loss of which confers a range of pleiotropic phenotypes. Elongator in higher eukaryotes is required for normal growth and development and a mutation in the largest subunit of human Elongator (Elp1) causes familial dysautonomia, a severe recessive neuropathy. Elongator promotes addition of mcm^5^ and ncm^5^ modifications to uridine in the tRNA anticodon ‘wobble’ position in both yeast and higher eukaryotes. Since these modifications are required for the tRNAs to function efficiently, a translation defect caused by hypomodified tRNAs may therefore underlie the variety of phenotypes associated with Elongator dysfunction. The Elp1 carboxy-terminal domain contains a highly conserved arginine/lysine-rich region that resembles a nuclear localization sequence (NLS). Using alanine substitution mutagenesis, we show that this region is essential for Elongator's function in tRNA wobble uridine modification. However, rather than acting to determine the nucleo-cytoplasmic distribution of Elongator, we find that the basic region plays a critical role in a novel interaction between tRNA and the Elp1 carboxy-terminal domain. Thus the conserved basic region in Elp1 may be essential for tRNA wobble uridine modification by acting as tRNA binding motif.

## Introduction

Elongator is a six-subunit protein complex (Elp1–6) that was first discovered in *Saccharomyces cerevisiae* (Otero *et al*., 1999) but which is present in all eukaryotes. A mutation in the *IKBKAP* gene encoding the human homologue of Elongator's largest subunit (Elp1) causes Familial Dysautonomia, a severe recessive neurodevelopmental disease (Anderson *et al*., 2001; Slaugenhaupt *et al*., 2001), while the human homologues of *ELP3* and *ELP4* have also been linked to the neurological disorders amyotrophic lateral sclerosis and rolandic epilepsy respectively (Simpson *et al*., 2009; Strug *et al*., 2009). In mice deletion of *ELP1* is embryonic lethal, suggesting that Elongator function is required during embryogenesis (Chen *et al*., 2009b) and in other higher eukaryotes, loss of Elongator function is associated with a range of developmental and growth defects (Nelissen *et al*., 2005; Chen *et al*., 2009a; Mehlgarten *et al*., 2010; Singh *et al*., 2010; DeFraia and Mou, 2011; Walker *et al*., 2011).

In budding yeast, the genes encoding Elongator are non-essential but when deleted lead to a common set of phenotypes (Otero *et al*., 1999; Frohloff *et al*., 2001; Li *et al*., 2009), one of which is resistance to zymocin, a protein toxin secreted by the yeast *Kluyveromyces lactis* (Frohloff *et al*., 2001). Zymocin is a tRNA anticodon nuclease that cleaves tRNA^Glu^(UUC) and to a lesser extent tRNA^Lys^(UUU) and tRNA^Gln^(UUG), three tRNAs that contain 5-methoxycarbonymethyl-2-thiouridine (mcm^5^s^2^U) in the anticodon wobble position (Lu *et al*., 2005). The mcm^5^s^2^U modification is essential for substrate recognition by zymocin, which targets tRNA^Glu^(UUC) for cleavage on the 3′ side of the wobble position (Lu *et al*., 2005). Elongator mutant strains lack the 5-methyoxycarbonylmethyl (mcm^5^) and 5-carbamoylmethyl (ncm^5^) modifications on wobble uridine-containing tRNAs and in the case of the zymocin substrate tRNAs, absence of the mcm^5^ moiety protects them from recognition and cleavage by zymocin (Huang *et al*., 2005). The Elongator complex is therefore required for an early step in synthesis of the mcm^5^ and ncm^5^ modifications on wobble uridine-containing tRNAs (Huang *et al*., 2005). In yeast, 11 out of 42 tRNAs carry these Elongator-dependent modifications, which are required for efficient decoding at the wobble position during translation (Huang *et al*., 2005; Agris, 2008; Johansson *et al*., 2008). Despite the variety of disparate roles that have been proposed for Elongator (Otero *et al*., 1999; Wittschieben *et al*., 1999; Rahl *et al*., 2005; Creppe *et al*., 2009), the major role of Elongator in yeast is now thought to be in tRNA wobble uridine modification: increased expression of tRNA^Lys^(UUU) and tRNA^Gln^(UUG) is sufficient to rescue the majority of *elp* mutant phenotypes tested so far (Esberg *et al*., 2006; Chen *et al*., 2011), providing strong evidence that these mutant phenotypes are caused by a common translational defect. Like Elongator itself, its requirement for tRNA wobble uridine modification is also conserved in worms, plants and mice (Chen *et al*., 2009a; Mehlgarten *et al*., 2010; Lin *et al*., 2013).

The Elongator holocomplex is currently thought to exist as a dodecamer containing two copies of each of its six subunits. Elp4, Elp5 and Elp6 each contain a common RecA-like NTPase fold and assemble into a heterohexameric ring structure with which two Elp1–3 heterotrimers have been proposed to associate (Glatt *et al*., 2012; Glatt and Muller, 2013). While it is not yet known whether Elongator acts directly in the catalysis of the tRNA modifications, Elp3 contains two functionally important enzymatic domains that may be involved; an N-terminal Radical *S*-adenosylmethionine (SAM)-like domain (Chinenov, 2002) and a C-terminal GNAT-type histone acetyltransferase (HAT) like domain (Wittschieben *et al*., 1999), both of which are essential for tRNA modification (Huang *et al*., 2005; Chen *et al*., 2011). The combination of these two functional domains in Elp3 is highly conserved and enzymes containing Radical SAM domains have already been implicated in other RNA modification reactions (Agarwalla *et al*., 2002; Pierrel *et al*., 2004; Anton *et al*., 2008; Yan *et al*., 2010; Grove *et al*., 2011). In addition, the Elongator accessory protein Kti12, which is related to *O*-phosphoseryl-tRNA^Sec^ kinase (PSTK; Sherrer *et al*., 2008), is also required for Elongator-dependent tRNA wobble uridine modification (Butler *et al*., 1994; Frohloff *et al*., 2001; Fichtner *et al*., 2002a) although its role is currently unknown.

Elp1 is the largest Elongator subunit and has been thought to act as a core scaffold, since interactions of Elp2 and Elp3 with each other and with components of the Elp4–6 subcomplex are lost when Elp1 is deleted (Fichtner *et al*., 2002b; Frohloff *et al*., 2003). Budding yeast Elp1 contains an Arg/Lys-rich basic region in the C-terminal domain that was previously noted as a putative nuclear localization sequence (NLS: Fichtner *et al*., 2002b, Fichtner *et al*., 2003). Since tRNA anticodon modifications are generally thought to occur in the cytoplasm (Hopper and Phizicky, 2003; Huang *et al*., 2005) and it is known that Elp1 is predominantly cytoplasmic in both yeast and human cells (Kim *et al*., 2002; Pokholok *et al*., 2002; Huh *et al*., 2003), it was unclear why the Elongator complex might require a nuclear import sequence. We therefore set out to investigate the importance of the Elp1 basic region in terms of Elongator function and have found that it mediates the binding of tRNA to Elp1, suggesting a critical role for the Elp1 C-terminal domain in the binding of tRNA to the Elongator complex.

## Results

### A highly conserved basic region in Elp1 is essential for its function

**Fig. 1:**
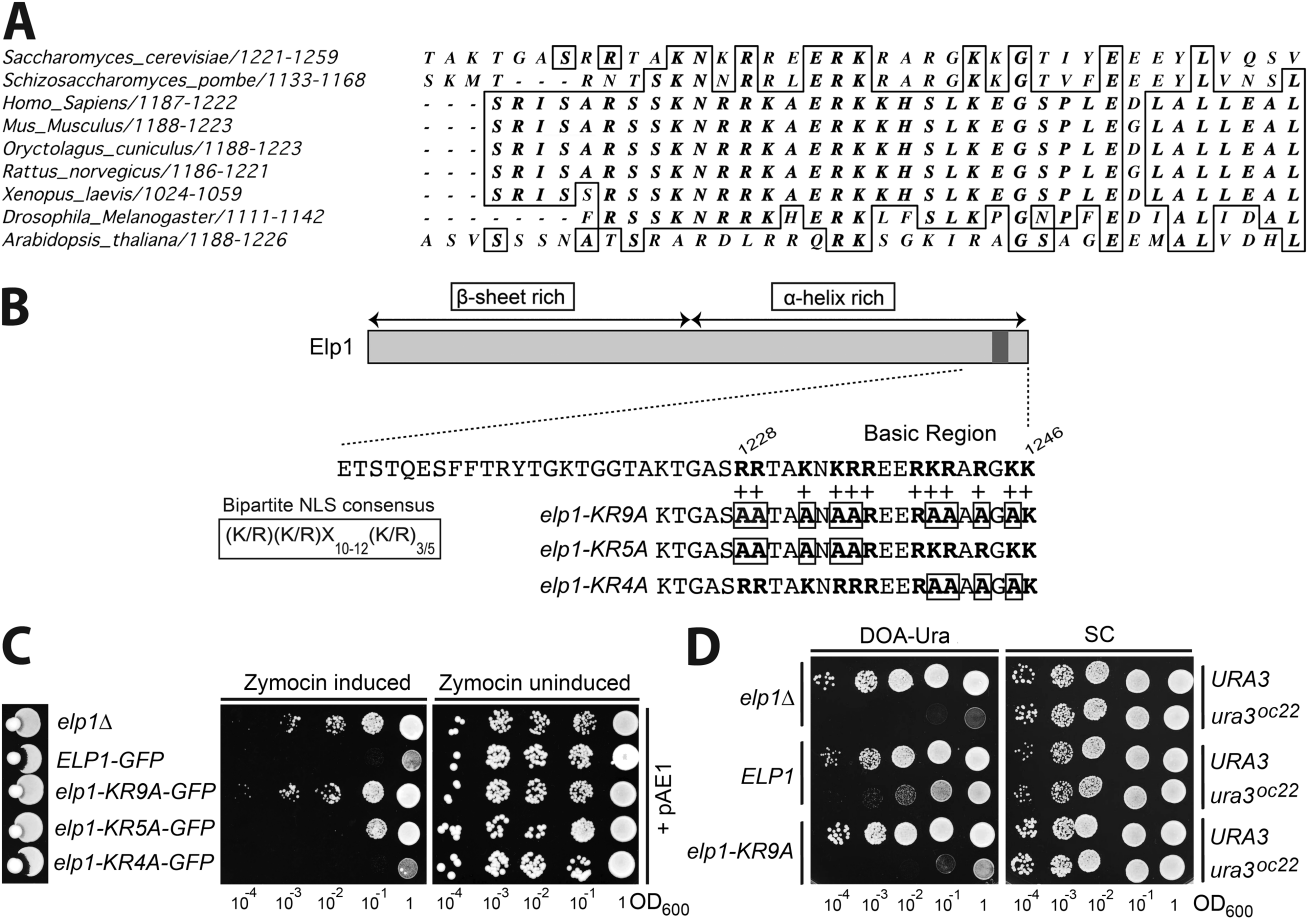
A highly conserved basic region in Elp1 is essential for Elongator function. A. Multiple sequence alignment of the Elp1 subunit of Elongator from various eukaryotic organisms showing a portion of the Elp1 C-terminal domain including the basic region to highlight its conservation. Alignment was carried out using Clustal Omega (http://www.ebi.ac.uk/Tools/msa/clustalo/). Sequences used were as follows with accession numbers in brackets; *S. cerevisiae* (Q06706), *S. pombe* (O59704), *H. sapiens* (O95163), *M. musculus* (Q7TT37), *O. cuniculus* (Q8WND5), *R. norveigus* (Q8VHU4), *X. laevis* (Q2TAQ1), *D. melanogaster* (Q9VGK7), *A. thaliana* (Q9FNA4). B. Schematic of Elp1 highlighting the Arg/Lys rich basic region (basic residues in bold) and alanine substitution mutations (boxed) made within this region. Mutations were made across the entire Elp1 basic region (*elp1–KR9A*) and within the first and second halves of the region (*elp1–KR5A* and *elp1–KR4A* respectively). For comparison the consensus sequence for a bipartite NLS is also shown. The indicated structural predictions were made using PSIPRED (Buchan *et al*., 2013). C. Zymocin sensitivity assays of strains containing GFP tagged wild-type *ELP1* or *elp1* basic region mutants at the *ELP1* genomic locus. Wild-type (YRDS253) and *elp1Δ* (YRDS250) served as controls for zymocin sensitivity and resistance respectively. Left panel, Eclipse assays on YPAD medium measuring growth inhibition adjacent to a colony of a zymocin-secreting *K. lactis* strain. Centre and right panels, growth of equivalent 10-fold serial dilutions (indicating OD_600_) of the indicated strains containing pAE1, which expresses the zymocin γ subunit under control of a galactose inducible promoter. Strains were grown on YPARG (centre panel: zymocin induced) and YPAD (right panel: zymocin uninduced). D. Growth of equivalent 10-fold serial dilutions of the indicated strains containing chromosomal copies of *ELP1* or *elp1–KR9A* together with the *SUP4 ochre* suppressor and either wild-type *URA3* (as a control) or the *ura3^oc22^ ochre* allele. *SUP4* can read through the premature stop codon in *ura3^oc22^* to allow growth in the absence of uracil on DOA-Ura medium but efficient suppression requires mcm^5^ modification of the *SUP4* tRNA at the wobble uridine position, as shown by comparing growth of the *ELP1* and *elp1Δ ura3^oc22^* strains. Growth of the same strains on synthetic complete (SC) medium without uracil selection is shown as a control.

The yeast Elp1 C-terminal domain contains an Arg/Lys-rich basic region that is highly conserved across eukaryotes (Fig. 1A), implying that it could be functionally important. This region had previously been noted to resemble a putative bipartite NLS (Fichtner *et al*., 2003), which has the classical consensus sequence (K/R)(K/R)X_10–12_(K/R)_3/5_ (Lange *et al*., 2007; Kosugi *et al*., 2009). To study the functional significance of this conserved basic region a series of mutations were designed throughout its sequence. A mutant allele (*elp1–KR9A*) was generated in which nine of the basic residues were substituted by alanine, as well as *elp1–KR5A* and *elp1–KR4A* alleles containing alanine substitutions in the first and second halves of the basic region respectively (Fig. 1B). All three mutants were incorporated into the genomic *ELP1* locus using the ‘*delitto perfetto’* approach.

Eclipse assays, in which sensitivity to *K. lactis* zymocin is scored by formation of a halo of growth inhibition around a colony of *K. lactis*, are a convenient method of assessing Elongator function because loss of Elongator-dependent tRNA modification confers resistance to this toxin (Huang *et al*., 2005). Figure 1C (left panel) shows that while strains with wild-type Elongator were sensitive to zymocin as expected, the *elp1Δ* control, *elp1–KR9A* and *elp1–KR5A* mutant strains were all resistant to zymocin, indicating that they are defective in Elongator function. In contrast, the *elp1–KR4A* mutant showed sensitivity to zymocin (Fig. 1C, left panel). To gain a more quantitative readout of Elongator function in the three mutants, a plasmid (pAE1) expressing the toxic γ subunit of zymocin under control of a galactose inducible promoter (Butler *et al*., 1994) was introduced into each strain and then intracellular expression of the γ subunit induced by growth on galactose-containing medium. Figure 1C (centre and right panels) shows that the *elp1–KR9A* mutant was fully resistant to intracellular zymocin expression in comparison with an *elp1Δ* strain that is completely lacking Elongator function. However, the *elp1–KR5A* strain showed a lower level of resistance while the *elp1–KR4A* mutant appeared as sensitive as the wild-type *ELP1* control. Taken together, these results indicate that *elp1–KR4A*, *elp1–KR5A* and *elp1–KR9*A show progressive reduction in zymocin sensitivity, with *elp1–KR9A* having the strongest resistance phenotype, comparable to that of the *elp1Δ* null strain. Although *elp1–KR4A* had little effect on its own, it clearly enhanced the defect of *elp1–KR5A* when the two sets of mutations were combined (*elp1–KR9A*) and since the *elp1–KR9A* mutant shows the most severe loss of function, the entire basic region must contribute to Elongator function.

To provide an additional readout of Elongator function, we next employed an assay that monitors readthrough of a *ura3 ochre* allele (*ura3^oc22^*) by the *SUP4 ochre* suppressor tRNA^Tyr^(UUA), which depends on Elongator-dependent modification of its wobble uridine residue for efficient UAA codon readthrough (Huang *et al*., 2005). Consequently, only strains with functional Elongator can efficiently suppress *ura3^oc22^* and grow well on medium lacking uracil, while strains that have compromised Elongator function and therefore reduced levels of mcm^5^U tRNA modification show strongly reduced growth on medium lacking uracil (DOA-Ura). The difference in such growth between wild-type *ELP1 SUP4 ura3^oc22^* and *elp1Δ SUP4 ura3^oc22^* strains indicates the extremes of phenotype against which other mutant *elp1* alleles can be compared. Figure 1D shows that the *elp1–KR9A* mutant and *elp1Δ* control strains conferred comparable defects in *SUP4 ochre* suppression, confirming that *elp1–KR9A* is largely lacking in Elongator function. Taken together, these results indicate that the Elp1 basic region is essential for Elongator-dependent tRNA modification function of Elongator.

### Elp1 basic region mutations do not alter its nucleo-cytoplasmic distribution

The yeast Elp1 basic region has been previously suggested to resemble a bipartite NLS and when fused to GFP, the Elp1 C-terminal domain (CTD) can direct import of the fusion protein into the nucleus (Fichtner *et al*., 2003). In spite of this, Elp1 has been reported to be mainly cytoplasmic in both yeast and human cells (Kim *et al*., 2002; Pokholok *et al*., 2002; Huh *et al*., 2003), while other work has shown that addition of a *bona fide* NLS onto the Elp1 C-terminus restricts Elp1 to the nucleus and causes loss of Elongator function, consistent with a cytoplasmic role for the complex (Rahl *et al*., 2005). However, the potential role of this region in determining the distribution of Elongator between the nucleus and the cytoplasm has not yet been fully addressed and in order to understand why Elp1's basic region is essential for Elongator function, we therefore wished first to exclude any roles it might have in determining the subcellular distribution of Elongator.

**Fig. 2:**
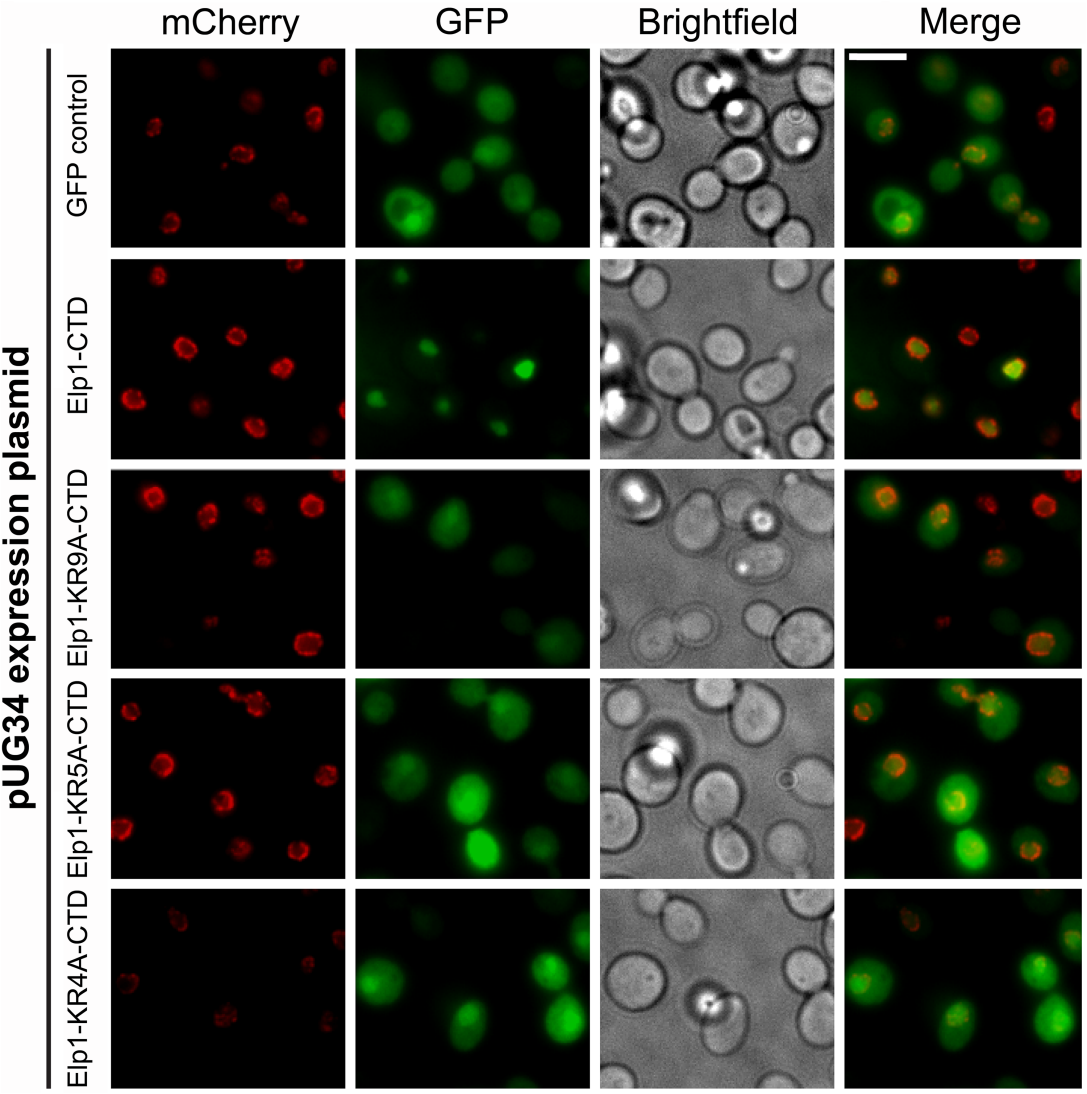
The Elp1 C-terminal domain can drive nuclear import of GFP dependent on the conserved basic region. Representative images of cells expressing Nic96-4mCherry (red) to indicate the nuclear periphery (YRDS84) containing either pUG34 (GFP control) expressing GFP or its derivative plasmids expressing GFP fused at its C-terminus to residues 1181–1349 from wild-type (Elp1–CTD) or the mutant (Elp1–KR4A-CTD, Elp1–KR5A–CTD, Elp1–KR9A–CTD) versions of Elp1 shown in Fig. 1 (green). Cells were grown to log phase in DOA-Met-His medium to induce GFP expression and then fixed for imaging. Both GFP (27 kDa) and the fusion proteins (47 kDa) are expected to be present in both the nucleus and cytoplasm in the absence of active nuclear localization signals as they are small enough to enter the nucleus by diffusion. CTD, C-terminal domain; scale bar: 5 μm.

In agreement with earlier work (Fichtner *et al*., 2003), we found that the Elp1 C-terminal domain (amino acids 1181–1349) was capable of driving nuclear import of GFP and that this was dependent specifically on the basic region. Thus GFP tagged with the wild-type Elp1 C-terminus was concentrated exclusively in the cell nucleus (Fig. 2), while the *elp1–KR9A*, *elp1–KR5A* and *elp1–KR4A* mutants all showed diffuse localization of GFP throughout the cell that was comparable to that of GFP alone, suggesting that when the basic region is mutated the C-terminus can no longer influence GFP localization. The basic region in the Elp1 carboxy-terminal domain therefore has the potential to drive nuclear import.

**Fig. 3:**
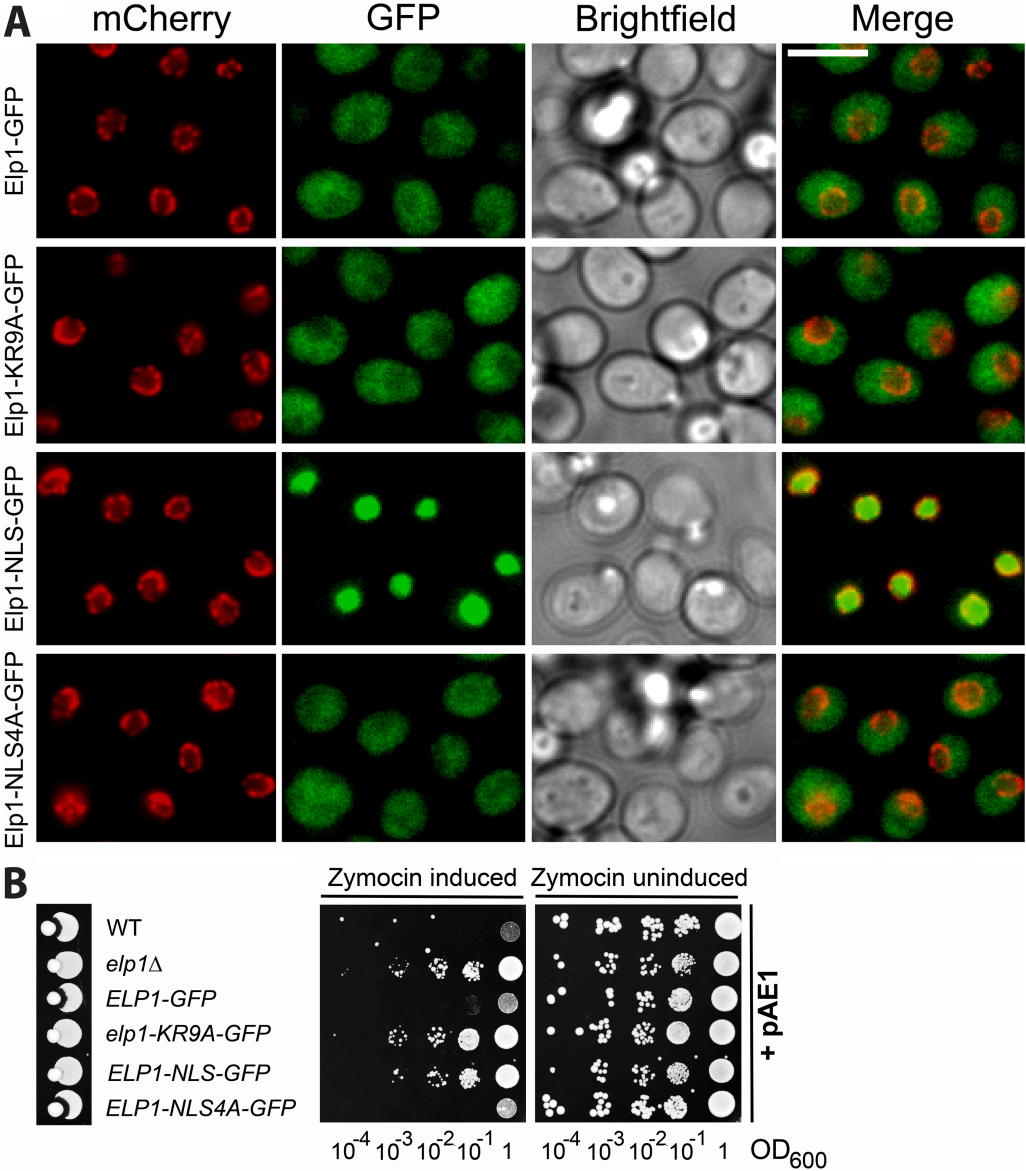
The nucleo-cytoplasmic distribution of Elp1 is unaffected by basic region mutations. A. Representative images of strains expressing Nic96-4mCherry (red) together with GFP-tagged wild-type Elp1, Elp1–KR9A, Elp1–NLS or Elp1–NLS4A (green). Elp1–NLS contains the NLS from Cbp80 and Elp1–NLS4A contains a mutant version containing 4 alanine substitutions, inserted in each case between the Elp1 and GFP sections of the fusion protein. Cells were grown to log phase in YPAD medium and then fixed for imaging. Scale bar: 5 μm. B. Zymocin sensitivity assays of the indicated strains as described in the legend to Fig. 2. Wild-type (WT, BY4741) and *elp1Δ* (YRDS250) served as controls for zymocin sensitivity and resistance respectively.

However, when the distribution of wild-type full length Elp1 between the nucleus and cytoplasm was examined using strains with GFP-tagged Elp1 and mCherry-tagged Nic96 (to define the nuclear boundary), localization of wild-type Elp1–GFP was largely cytoplasmic (Fig. 3A) in agreement with previous reports (Pokholok *et al*., 2002; Huh *et al*., 2003; Tkach *et al*., 2012), but with low levels of Elp1–GFP fluorescence in the nucleus. Furthermore, when the localization of the Elp1–KR9A–GFP mutant was also assayed it showed no difference in the nucleo-cytoplasmic distribution of Elp1 in comparison with wild-type Elp1–GFP (Fig. 3A). Thus while the basic region of Elp1 can drive nuclear import in isolation, in the context of the whole polypeptide this does not appear to be functionally relevant and the apparent nucleo-cytoplasmic distribution of Elp1 was unaffected by the basic region mutation. The *elp1–KR5A* and *elp1–KR4A* mutants also showed no differences in Elp1 localization (data not shown). The fact that the *elp1–KR4A* mutant was almost normal in terms of Elongator function in comparison with the defective *elp1–KR9A* allele (Fig. 1) and yet the C-terminal domain from either mutant protein was equally compromised in its ability to drive nuclear import of GFP (Fig. 2) is consistent with the role of this region being unrelated to potential NLS function. Finally, we confirmed that when the NLS from Cbp80 was inserted into our Elp1–GFP construct that Elp1–NLS–GFP localized exclusively to the nucleus (Fig. 3A) and that this was associated with complete loss of Elongator-dependent tRNA modification as assayed by zymocin resistance (Fig. 3B). In contrast, when four basic amino acids in the Cbp80 NLS sequence were mutated, Elp1 localization reverted to a mainly cytoplasmic distribution and full sensitivity to zymocin was restored (Fig. 3). This extends the previous findings (Rahl *et al*., 2005) by showing that Elongator's tRNA wobble uridine modification function, as assayed by zymocin sensitivity, is abolished when Elp1 is trapped in the nucleus. This supports a tRNA modification role for Elongator that requires cytoplasmic rather than nuclear localization. Taken together, these data are therefore consistent with the functional importance of the Elp1 basic region being not through acting as a determinant of nucleo-cytoplasmic localization but through playing some other role.

### The Elp1 basic region mutation does not disrupt Elongator complex assembly but leads to subtle differences in subunit association

Recent work has suggested that the dodecameric Elongator holocomplex assembles such that a heterohexameric ring of Elp4–6 bridges two subcomplexes of Elp1–Elp3 (Glatt *et al*., 2012). Furthermore, the accessory protein Kti12 binds to the preassembled Elongator complex and is also required for its tRNA modification function (Butler *et al*., 1994; Frohloff *et al*., 2001; Fichtner *et al*., 2002a). It was therefore possible that the Elp1 basic region could act as a protein interaction domain that is required for assembly of the holocomplex. To examine Elongator complex assembly in the *elp1–KR9A* basic region mutant, the interaction of GFP tagged Elp1 and Elp1–KR9A with other HA tagged Elongator complex members was tested. Representative members of the two subcomplexes of Elongator, Elp3 and Elp5 respectively, as well as Kti12, were HA tagged in strains expressing GFP tagged Elp1 or Elp1–KR9A. As a control for the specificity of these protein–protein interactions, Elp3, Elp5 and Kti12 were also HA tagged in a strain expressing Arg4–GFP, a protein with no known connection to Elongator but reported to be present at similar levels.

**Fig. 4:**
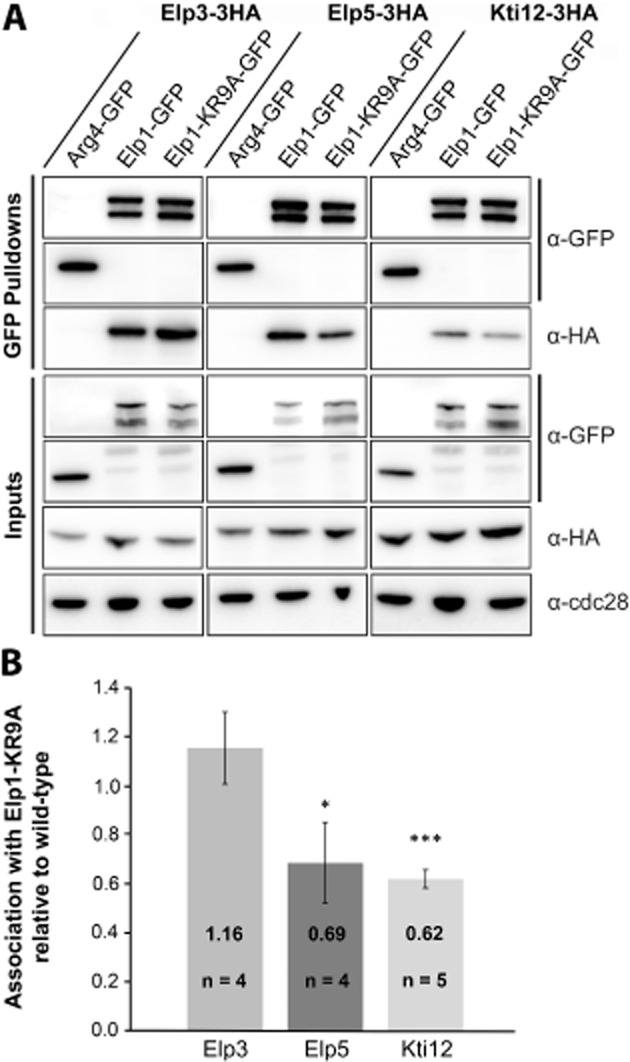
The *elp1–KR9A* mutation does not disrupt Elongator complex assembly but causes subtle differences in association with Elp5 and Kti12. A. Western blot analysis of inputs and GFP–Trap immunoprecipitates of Elp1–GFP and Elp1–KR9A–GFP from strains expressing HA-tagged Elp3, Elp5 or Kti12. All samples were analysed with anti–GFP and anti-HA. Input samples were also analysed using anti-Cdc28 as a loading control. Arg4–GFP was confirmed to have similar pulldown efficiency to Elp1–GFP and was used as a control to demonstrate that interaction between Elp1 and the various HA tagged complex members was specific. All pulldown samples were analysed from the same blot. It should be noted that Elp1 is always observed by Western blot analysis as both a full-length and a faster migrating form (truncated at its N-terminus) that may be a degradation product or serve an as yet unknown function (Fichtner *et al*., 2003). B. Quantification of co-immunoprecipitation efficiency. Immunoprecipitation of HA-tagged proteins was quantified by densitometry of the HA tag signals and normalized using the Elp1–GFP signals across the indicated number of replicates (n), setting the value for the wild-type strain in each case to 1.0. Error bars represent the standard deviation of the mean and the significance of the differences was analysed using a one way ANOVA, with ‘*’ indicating a *P*-value of < 0.05 (significant) and ‘***’ indicating a *P*-value of < 0.001 (very highly significant). Any small differences in Elp3 association were not statistically significant when analysed in this manner.

GFP–Trap immunoprecipitations of Elp1–GFP and Elp1–KR9A–GFP were assayed for association with HA tagged Kti12, Elp3 and Elp5 and these were each present as co-precipitants under physiological salt conditions (Fig. 4A). This confirmed both that the Elp1–3 sub complex was intact and that interactions with Elp5 and Kti12 are not abolished by the basic region mutation, and so the Elp1 basic region is not required for the overall gross assembly of the Elongator complex. However, when these interactions were analysed carefully by densitometry, Elp1–KR9A–GFP was consistently found associated with ∼ 30% less Elp5-3HA and ∼ 40% less Kti12-3HA than the wild-type Elp1–GFP protein (Fig. 4B). These minor interaction defects are highly consistent and statistically significant. In contrast, Elp3 levels and the interaction between Elp1–KR9A–GFP and Elp3-3HA appeared largely unchanged (Fig. 4B), consistent with earlier work in which Elp3 was found to become very unstable in strains where it could not interact with Elp1 (Petrakis *et al*., 2004). The small defects in Kti12 and Elp5 interactions with the Elp1–KR9A mutant may reflect the fact that Elongator is no longer functional. For example, if the complex needs to shuttle between multiple forms and is stalled due to the basic region mutations, this may lead to reduced complex stability and subsequently affect the interaction with other subunits.

**Fig. 5:**
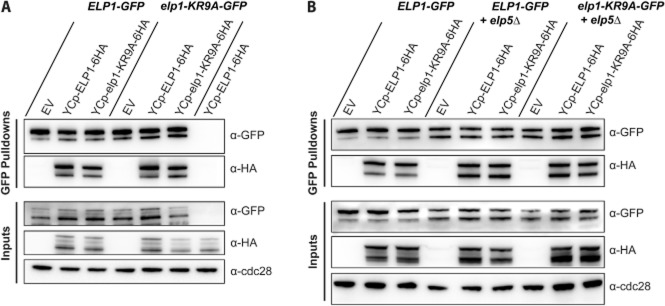
Self-association of Elp1 is unaffected by the *elp1–KR9A* mutation and is independent of the assembly of the Elp4–6 hexamer. A. Western blot analysis of inputs and GFP–Trap immunoprecipitates of Elp1–GFP and Elp1–KR9A–GFP from strains containing empty vector (EV), YCplac111–*ELP1*–6HA or YCplac111–*elp1–KR9A*–6HA expressing HA-tagged wild-type or mutant Elp1 respectively. All samples were analysed by Western blotting with anti–GFP and anti-HA. Inputs were also analysed using anti-Cdc28 as a loading control. B. Western blot analysis of inputs and GFP–Trap immunoprecipitates of Elp1–GFP and Elp1–KR9A–GFP from strains containing empty vector (EV), YCplac111–*ELP1*–6HA or YCplac111–*elp1–KR9A*–6HA expression plasmids either with or without deletion of the *ELP5* gene (*elp5Δ*).

Since it was recently shown that Elongator forms a dodecamer containing two copies each of its six subunits (Glatt *et al*., 2012), it was possible that Elp1–KR9A could be defective in assembly into this higher order complex. To test this, YCp–*ELP1*–6HA and YCp–*elp1–KR9A*–6HA expression plasmids were transformed into *ELP1*–GFP and *elp1–KR9A*–GFP strains to generate cells in which two differentially tagged copies of Elp1 were present. The *ELP1*–GFP strain remained sensitive to zymocin following introduction of YCp–*ELP1*–6HA, confirming that the increased *ELP1* copy number did not cause significant loss of function (data not shown). Wild-type Elp1–GFP and Elp1–KR9A–GFP were immunoprecipitated using GFP–Trap and assayed for co-precipitation of the 6HA-tagged versions of Elp1 (Fig. 5A). GFP-tagged wild-type Elp1 co-precipitated Elp1–6HA as previously reported (Glatt *et al*., 2012) and could also associate to a similar extent with Elp1–KR9A–6HA. In the reciprocal experiment, GFP tagged Elp1–KR9A also co-precipitated both Elp1–6HA and Elp1–KR9A–6HA, again with no obvious defect in the interaction between any of the different combinations. Thus there is no apparent defect in Elp1 self-association within the Elongator complex due to the Elp1 basic region mutation.

Since there was no obvious deficiency in Elp1–KR9A self-association despite its reduced interaction with Elp5, it seemed possible that Elp1 might associate independently of interaction between Elp1–3 subcomplexes and the Elp4–6 hexamer. To test this, the above immunoprecipitations were repeated in the context of an *elp5* deletion, which would prevent assembly of the Elp4–6 hexamer (Glatt *et al*., 2012). Fig. 5B shows that neither wild-type Elp1–GFP nor Elp1–KR9A–GFP showed any defect in self-association in the absence of Elp5, suggesting that this interaction occurs independently and does not require bridging of Elp1–3 subcomplexes by an Elp4–6 hexamer. Taken together, mutation of the Elp1 basic region does not disrupt the overall assembly of Elongator, although it leads to small reductions in the association of Elp1 with Elp5 and Kti12 that may be due to loss of Elongator function.

### The basic region in Elp1 is required for the binding of tRNA to the Elp1 C-terminal domain

**Fig. 6:**
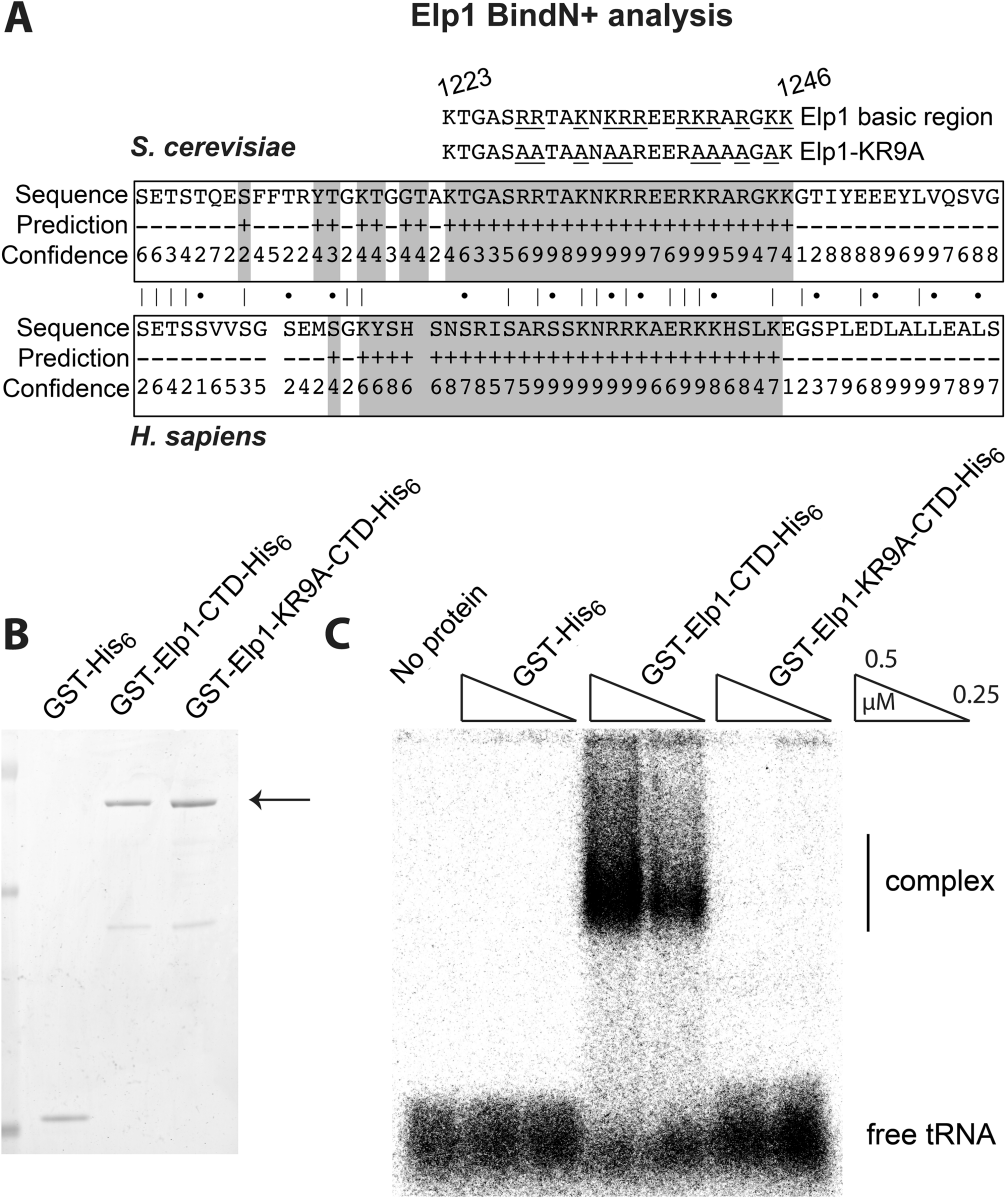
The Elp1 basic region is predicted to bind RNA and is required for formation of a complex with yeast tRNA. A. Analysis for predicted RNA binding sites within the *S. cerevisiae* and *H. sapiens* Elp1 protein sequence using BindN+ (http://bioinfo.ggc.org/bindn+/) showing the high confidence prediction of the basic region as a putative RNA binding region (confidence is scored on a scale of 1–9 with 9 as highest confidence). The locations of the Elp1 basic region and the *elp1–KR9A* alanine substitution mutations are shown for comparison. Overall conservation between the human and yeast sequences is also indicated (|, identity; •, similar residues). B. Recombinant Elp1 and Elp1–KR9A C-terminal domains (CTDs). Elp1 CTDs with N-terminal GST and C-terminal His_6_ tags were expressed and purified from *E. coli* along with GST-His_6_ as a control. The corresponding proteins were analysed on a 4–12% polyacrylamide gel and stained with instant blue. The GST–Elp1–CTD-His_6_ fusion proteins are indicated by an arrow. C. Electrophoretic mobility shift assay (EMSA) using ∼ 1 nM ^32^P-labelled yeast tRNA and the indicated concentrations of recombinant GST, GST–Elp1 and GST–Elp1–KR9A fusion proteins shown in (B). Binding reactions were separated on a 1.5% native agarose gel and analysed by autoradiography. A complex was formed between tRNA and the wild-type Elp1 CTD but not with the Elp1–KR9A CTD.

The conserved basic region in Elp1 is essential for tRNA wobble uridine modification but despite being capable of functioning as an NLS, it does not appear to influence the distribution of the Elp1 between the nucleus and cytoplasm and is not required for overall assembly of the Elongator complex. In keeping with Elongator's role in tRNA wobble uridine modification, it was recently shown by electrophoretic mobility shift assay (EMSA) that the Elp4–6 hexameric subcomplex of Elongator directly binds tRNA^Glu^(UUC), consistent with a direct role for Elongator in tRNA binding and subsequent modification (Glatt *et al*., 2012). Since the basic region in Elp1 was clearly important for Elongator function we hypothesized that it could be involved in binding tRNA molecules to the Elp1–3 subcomplex for wobble uridine modification, given that the Elp3 subunit within this subcomplex is the most likely candidate for carrying out the modification reaction. Elp1 was therefore analysed for potential RNA binding sites using the binding prediction program BindN+, which predicts RNA binding regions from primary sequence based on the properties of RNA binding residues from known structures (Wang and Brown, 2006). This analysis highlighted the conserved carboxy-terminal basic region in both yeast and human Elp1 as being likely to bind RNA (Fig. 6A), consistent with the notion that it may be required for tRNA binding. Such a role might therefore explain the profound consequences of the *elp1–KR9A* allele on Elongator function.

To address a potential role in tRNA binding, the C-terminal domains (CTD) of Elp1 and Elp1–KR9A were expressed and purified from *E. coli* using N-terminal GST and C-terminal His_6_ tags (Fig. 6B). As a control, GST-His_6_ was also purified in parallel. An electrophoretic mobility shift assay (EMSA) was carried out using these fusion proteins and [^32^P]-tRNA, resolving the tRNA-protein binding reactions by agarose gel electrophoresis. Figure 6C clearly shows that the wild-type Elp1 fusion protein was able to shift the radiolabelled tRNA to a much slower migrating form, indicating formation of a tRNA-protein complex that was absent both in the GST control and in the Elp1–KR9A binding reactions (Fig. 6C). The Elp1–CTD can therefore bind tRNA, but this ability is lost when the basic region is mutated in Elp1–KR9A.

**Fig. 7:**
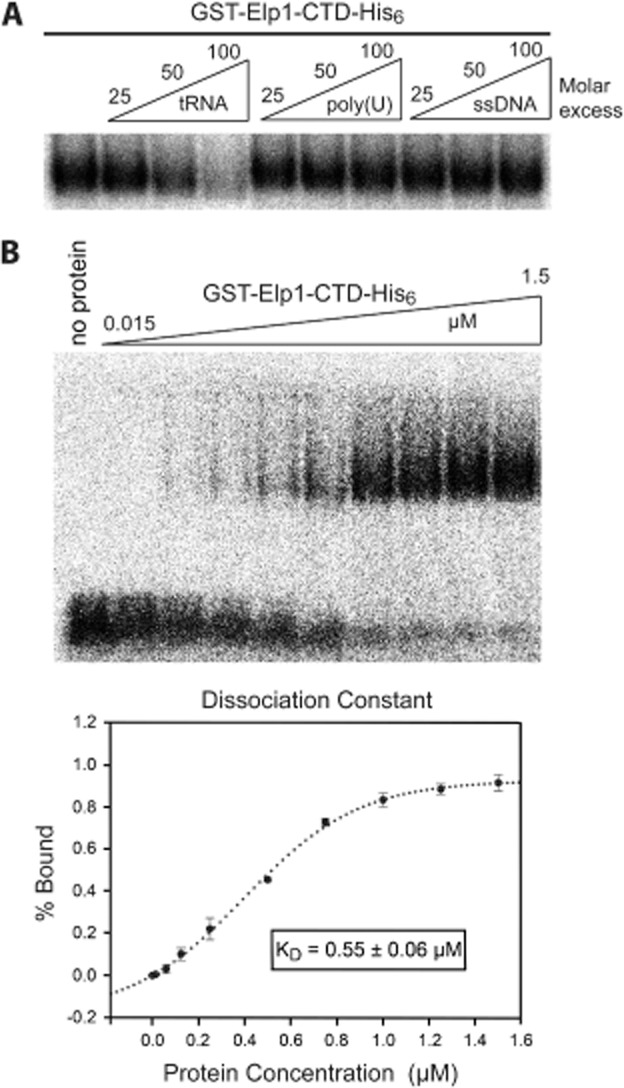
Binding of the Elp1 C-terminal domain to tRNA occurs with a dissociation constant in the low micromolar range and is not competed by poly(U) or ssDNA. A. Electrophoretic mobility shift assay (EMSA) using ∼ 1 nM ^32^P-labelled yeast tRNA, 0.4 μM recombinant Elp1 C-terminal domain and the indicated molar excess of unlabelled yeast tRNA, poly(U) or ssDNA. B. EMSA using ∼ 0.1 nM ^32^P-labelled yeast tRNA and the indicated concentrations of recombinant Elp1 C-terminal domain. Bound and free tRNA bands were quantified from three replicate experiments and used to estimate a dissociation constant (K_D_) of 0.55 μM for the interaction by non-linear curve fitting (*f* = a*x^b^/[*c^b^* + *x^b^*]). The Hill coefficient (*b*) for the fitted curve was 1.76 ± 0.19. All binding reactions were separated on a 1.5% native agarose gel and analysed by autoradiography.

To test the specificity of the interaction between the Elp1–CTD and tRNA, competition binding experiments were carried out (Fig. 7A). While addition of unlabelled yeast tRNA at a 100-fold excess efficiently competed formation of the [^32^P]-tRNA–Elp1–CTD complex, addition of a similar excess of either poly(U) or ssDNA left complex formation between the tRNA probe and Elp1–CTD essentially unaffected. The Elp1 C-terminal domain therefore preferentially binds tRNA over poly(U) RNA or ssDNA. We also compared the ability of tRNA purified from either wild-type or *elp1Δ* mutant yeast (in which wobble uridine-containing tRNAs lack the Elongator-dependent mcm^5^ and ncm^5^ modifications) as competitors of [^32^P]-tRNA binding to the Elp1 CTD. However, no differences were found (data not shown), suggesting that there are unlikely to be very large differences in affinity resulting from whether or not the tRNA carries Elongator-dependent modifications.

To estimate the dissociation constant (K_D_) of the Elp1–tRNA complex, binding reactions were set up with [^32^P]-tRNA and increasing concentrations of wild-type Elp1–CTD, ranging from 0.015 to 1.5 μM (Fig. 7B). The % bound values were quantified, plotted against the protein concentration and fitted to a non-linear sigmoidal curve. A K_D_ value of 0.55 μM was estimated from determining the protein concentration that gave ∼ 50% binding and this value is similar to that which was reported for the interaction between tRNA^Glu^(UUC) and recombinant Elp4–6 hexamers (Glatt *et al*., 2012).

These data therefore demonstrate a novel role for the C-terminal domain of Elp1 in tRNA binding and show that Elp1–KR9A, in which the basic region of Elp1 is mutated, has lost the ability to form this complex. We therefore propose that this basic region in Elp1 functions as a conserved tRNA binding motif that is important for Elongator-dependent wobble uridine modification.

## Discussion

### A conserved basic region in Elp1 is essential for Elongator function

The Elp1 subunit of Elongator contains a conserved lysine/arginine-rich basic region in its carboxy-terminal domain and we have shown that mutation of this region leads to zymocin resistance and loss of Elongator-dependent *SUP4 ochre* suppressor function, phenotypes indicative of a defect in Elongator-dependent tRNA wobble uridine modification. Although the basic region can function as an NLS in isolation, in comparison with full length wild-type Elp1–GFP we found no difference in the distribution of the Elp1–KR9A mutant protein between the nucleus and cytoplasm. Thus in the context of the full-length Elp1 protein that can be assembled into Elongator the Elp1 basic region does not function as an NLS, leaving open the question of why this region is essential for Elongator-dependent tRNA modification. Our finding that the basic region mediates binding of tRNA to Elp1 that is resistant to competition by non-specific polynucleotides and that is dependent on the residues altered in the *elp1–KR9A* allele provides a likely answer to this question. We therefore propose that the conserved Elp1 basic region is involved in the interaction of tRNA with the Elongator complex.

### The Elp1 basic region constitutes a novel tRNA binding domain

Although a direct role for Elongator in tRNA modification remains to be demonstrated, it seems likely that this is the primary role of the complex at least in yeast (Huang *et al*., 2005; Esberg *et al*., 2006). Our finding that the Elp1 C-terminal domain can bind tRNA with high nanomolar affinity, together with the previous demonstration of direct tRNA binding by the Elp4–6 hexamer (Glatt *et al*., 2012), lends credence to the idea that Elongator is directly involved in the tRNA modification reaction. Our calculated K_D_ of ∼ 0.55 μM for interaction between tRNA and the Elp1 C-terminal domain and tRNA complex indicates an interaction of similar strength to that observed between tRNA and the Elp4–6 hexamer (K_D_ of 1.2–1.4 μM; Glatt *et al*., 2012). Interestingly, the Hill coefficient value calculated from our binding curves (1.76) is indicative of positive cooperativity, suggesting either that the Elp1–C-terminal region may interact with tRNA as a dimer or that each Elp1 molecule can bind > 1 tRNA, although further work would be needed to confirm this. Given an estimated 3.3 × 10^6^ tRNA molecules per yeast cell (Waldron and Lacroute, 1975) and a mean cell volume of 37 fl (Tyson *et al*., 1979) we can estimate that the cellular total tRNA concentration is of the order of ∼ 150 μM, which would imply that binding in the cell would be saturated, even if only the 11 out of 42 tRNA species that require Elongator-dependent tRNA modification (Johansson *et al*., 2008) are bound.

However, it seems likely that there is little binding selectivity by either the Elp1 C-terminal domain or the Elp4–6 hexamer for tRNAs containing modifiable wobble uridines. Thus the tRNA–Elp4–6 complex was still formed regardless of the identity of the base at the wobble position of the tRNA probe used (Glatt *et al*., 2012), while in our study it is clear that a large proportion of the tRNA probe, which was prepared using total yeast tRNA, is capable of binding to Elp1. Furthermore, we found no difference in the ability of tRNA isolated from wild-type or Elongator-deficient *elp1Δ* mutant yeast cells (in which wobble uridines would be unmodified) to compete for binding with the [^32^P]-tRNA probe (data not shown). Thus if Elongator does directly modify wobble uridine-containing tRNAs it appears likely that substrate specificity is not determined at the level of tRNA binding to the complex. This could be considered analogous to how ribosomes select aminoacyl-tRNAs during protein synthesis: any charged tRNA can enter the ribosomal A-site, but only one that has a cognate anticodon–codon interaction has its binding stabilized so that a new peptide bond can form. Future work will need to identify whether tRNA interaction with Elp1 or Elongator as a whole shows any specificity towards substrate tRNAs containing a uridine in the wobble position, why it now seems likely that both subcomplexes of Elongator can bind tRNA, and whether extra stability and specificity of tRNA binding could be imparted within the context of the holo–Elongator complex.

### Effects of *elp1–KR9A* on Elongator's protein–protein interactions

Since none of the Elongator protein–protein interactions tested were lost as a result of the *elp1–KR9A* mutation, including the self-association of Elp1, it is unlikely that the basic region of Elp1 constitutes a domain essential for complex assembly. However, we observed subtle reductions in the association of Elp1–KR9A with both Elp5 and Kti12 that may occur because the complex is non-functional. For example, if Elongator cycles between different forms in order to bind, modify and release tRNA molecules then a mutation that disrupts tRNA binding could stall the complex in a particular conformation in which certain protein–protein interactions are destabilized. The Elp4–6 hexamer can be easily dissociated from Elp1–3 (Winkler *et al*., 2001), and rather than forming a static structure might undergo cycles of conformational changes, as seen with other hexameric RecA-like NTPase complexes (Lyubimov *et al*., 2011). Assuming this is the case, reduced association of Elp5 with Elp1–KR9A may occur due to an arrest in the mechanism of action of Elongator that weakens the interaction between the two subcomplexes. The Kti12 accessory protein associates with the assembled holocomplex (Butler *et al*., 1994; Fichtner *et al*., 2002a) and also contains a highly conserved NTP binding domain (Fichtner *et al*., 2002a), suggesting it could cycle between different nucleotide bound forms during the process of tRNA modification. Since truncation of the Elp1 carboxy-terminus including the basic region (deletion of residues 1229–1349) still allowed the same level of Kti12 association as with full-length Elp1 (Fichtner *et al*., 2002b), it seems unlikely that the basic region is a major determinant of Kti12 interaction, consistent with the reduced association that we see being a secondary consequence of the *elp1–KR9A* mutation. A better understanding of the relationship between subunit interactions, Kti12 association and the mechanism of tRNA binding is required to understand the significance of these observations. Another exciting possibility is that tRNA binding and/or modification could be regulated by Elp1 phosphorylation, which is known to be correlated with Elongator function (Mehlgarten *et al*., 2009).

In summary, our work has shown that the Elp1 basic region mediates tRNA binding to Elp1 and it therefore forms a highly conserved tRNA binding motif in Elongator. This is the first evidence that Elp1, the largest subunit of Elongator, is involved in the interaction with tRNA and supports a direct role for the complex in tRNA modification.

## Experimental procedures

### Plasmids

Cloning was carried out using standard methods (Sambrook and Russell, 2001). pAE1, in which the zymocin γ subunit can be expressed from the *GAL1* promoter, is based on the YCp50 (*URA3*) plasmid pBM150 (Johnston and Davis, 1984) and has been described previously (Butler *et al*., 1991). Plasmids containing *elp1* mutations were generated by DNA2.0, Inc. from YCplac111–*ELP1*–6HA by replacing the relevant part of the gene with a synthesized *elp1* mutant sequence. YCplac111–*ELP1*–6HA was kindly provided by R. Schaffrath (University of Kassel) and consists of YCplac111 (Gietz and Akio, 1988) containing the *ELP1* open reading frame with 632 bp of upstream sequence, fused to a 6HA tag and followed by the *K. lactis IPP1* transcription terminator (D. Jablonowski and R. Schaffrath, unpublished).

For conditional expression of GFP fused to the Elp1 C-terminal domain (amino acids 1181–1349), the relevant region from YCplac111–*ELP1*–6HA or its mutant derivatives were amplified by PCR and cloned into the GFP tagging vector pUG34 (GenBank accession AF298784; a kind gift from Johannes Hegemann), thereby allowing expression of the GFP–Elp1 fusion proteins from the *MET25* promoter.

To generate templates for C-terminal tagging of Elp1 with an NLS sequence, a 462 bp DNA sequence (‘gBlock’; Integrated DNA technologies) was used to replace the plasmid sequence between endogenous PacI and BsrGI sites in pFA6a–GFP(S65T)–HIS3MX6 (Longtine *et al*., 1998) in order to include upstream of the GFP gene a sequence encoding the first 30 amino acids of yeast Cbp80 that contains a nuclear localization sequence (Izaurralde *et al*., 1995), generating pFA6a-NLS–GFP(S65T)–HIS3MX6. A mutant version of this construct [pFA6a-NLS4A–GFP(S65T)–HIS3MX6] containing four alanine substitution mutations to disrupt NLS function was similarly generated. For recombinant expression of Elp1, the region encoding the C-terminal half (amino acids 730–1349) was amplified by PCR so as to include a C-terminal His_6_ tag and cloned into pGEX4T-1 (GE Healthcare; GenBank accession U13853).

### Yeast strains and methods

Yeast strains used in this study are summarized in Table 1. Basic yeast methods, growth media, and routine recombinant DNA methodology were performed as previously described (Amberg *et al*., 2005). Haploid budding yeast strains were cultured in rich yeast-peptone-adenine-dextrose (YPAD) medium or the appropriate selective dropout media (DOA) at 30°C. Yeast transformations were carried out using the Lithium acetate/PEG method (Gietz and Woods, 2002). Eclipse assays were carried out as described (Kishida *et al*., 1996) using *Kluyveromyces lactis* (NCYC1368) and photographed after 1–2 days.

**Table 1:** Yeast strains

Strain	Genotype
*Kluyveromyces lactis*	
NCYC1368	prototroph pGLK2^+^ pGLK1^+^^a^
*Saccharomyces cerevisiae*	
BY4741	*MAT***a** *his3Δ1 ura3Δ0 leu2Δ0 met15Δ0*^b^
SBY128	BY4741 *his3Δ1*::pSB1(*URA3*, *SUP4*, *NatRMX*)
SBY132	BY4741 *his3Δ1::*pSB3 (*ura3^oc22^, SUP4*, *NatRMX*)
YRDS1	BY4741 *NIC96*-4mcherry*::NatRMX*
YRDS59	YRDS1 *ELP1*-L-GFP-*HIS3MX*^c^
YRDS84	BY4741 *NIC96*-4mcherry*::NatRMX lys2Δ0*
YRDS228	BY4741 *elp1::*CORE
YRDS229	YRDS59 *elp1::*CORE*-*L*-*GFP-*HIS3MX*
YRDS250	BY4741 *elp1Δ::HIS3MX*
YRDS253	YRDS229 *ELP1*-L-GFP-*HIS3MX*^d^
YRDS255	YRDS229 *elp1-KR9A*-L-GFP-*HIS3MX*^d^
YRDS259	SBY128 *elp1Δ::HIS3MX*
YRDS261	SBY132 *elp1Δ::HIS3MX*
YRDS270	YRDS228 *ELP1 his3Δ1*::pSB1(*URA3*, *SUP4*, *NatRMX*)^d^
YRDS272	YRDS228 *ELP1 his3Δ1::*pSB3 (*ura3^oc22^, SUP4*, *NatRMX*)^d^
YRDS278	YRDS228 *elp1-KR9A his3Δ1*::pSB1(*URA3*, *SUP4*, *NatRMX*)^d^
YRDS280	YRDS228 *elp1-KR9A his3Δ1::*pSB3 (*ura3^oc22^, SUP4*, *NatRMX*)^d^
YRDS317	YRDS229 *elp1-KR5A*-L-GFP-*HIS3MX*^d^
YRDS319	YRDS229 *elp1-KR4A*-L-GFP-*HIS3MX*^b^
YRDS439	YRDS1 *ARG4*-L-GFP-*HIS3MX*
YRDS461	YRDS253 *KTI12*-L-3HA-*KanMX*
YRDS463	YRDS253 *ELP3*-L-3HA-*KanMX*
YRDS467	YRDS255 *KTI12*-L-3HA-*KanMX*
YRDS474	YRDS253 *ELP5*-L-3HA-*KanMX*
YRDS482	YRDS255 *ELP3*-L-3HA-*KanMX*
YRDS487	YRDS255 *ELP5*-L-3HA-*KanMX*
YRDS526	YRDS439 *KTI12*-L-3HA-*KanMX*
YRDS528	YRDS439 *ELP5*-L-3HA-*KanMX*
YRDS537	YRDS439 *ELP3*-L-3HA-*KanMX*
YRDS539	YRDS1 *ELP1*-L-NLS4A-L-GFP-*HIS3MX*
YRDS544	YRDS1 *ELP1*-L-NLS-L-GFP-*HIS3MX*

a. National Collection of Yeast Cultures (http://www.ncyc.co.uk).

b. Brachmann *et al*. (1998).

c. ‘L’ indicates inclusion of a linker region (see *Experimental procedures*).

d. Created by ‘*delitto perfetto’* method of integration (see *Experimental procedures*).

Gene knockout and C-terminal tagging cassettes for transformation were generated as described previously (Longtine *et al*., 1998) unless otherwise indicated. In several cases, the forward primer was designed to incorporate a linker (encoding the amino acid sequence –GSAGSAAGSG–) between the end of the gene-specific sequence and the tag. To visualize the nuclear periphery in yeast cells, the nuclear pore complex component Nic96 was tagged with 4mCherry as outlined above, using pT909 as the template (a kind gift from Tomoyuki Tanaka, University of Dundee). Correct integration of gene knockouts or C-terminal tagging cassettes were confirmed by colony PCR and if appropriate by fluorescence microscopy and Western blotting.

The ‘*delitto perfetto’* method was used to introduce site-directed mutations into the genomic copy of *ELP1* (Storici and Resnick, 2006), using YCplac111–*ELP1*–6HA and its mutant derivatives as a template to generate the DNA fragments required to replace marker genes inserted at the point where the mutation was to be made. Correct integration of the mutant fragment was confirmed by DNA sequencing of a PCR product encompassing the sequences replaced by the mutant fragment.

### Monitoring Elongator function through efficiency of SUP4 ochre suppression

To monitor the efficiency of suppression by the Elongator-dependent *SUP4 ochre* suppressor tRNA^Tyr^(UUA) we constructed a plasmid carrying *SUP4* and a *ura3 ochre* allele (*ura3^oc22^*) that could be used to introduce both the tRNA and a suppressible allele into yeast cells. The *ura3^oc22^* mutation was generated by site-directed mutagenesis using the QuikChange® Multi Site-Directed Mutagenesis Kit (Stratagene), the *URA3* gene from YDpU (Berben *et al*., 1991) as template and a synthetic oligonucleotide carrying the desired *ochre* point mutation (5′-CATGTCGAAAGCTACATATAAG**T**AACGTGCTGCTACTCATCC-3′, mutational change is underlined). The mutated gene was moved as an 1126 bp NheI–BamHI fragment into the BamHI–XbaI interval of pRS303 (Sikorski and Hieter, 1989), following which a 270 bp fragment from pTC3 (Shaw and Olson, 1984) carrying *SUP4* was inserted into the unique BamHI site to generate pRS303-*URA3-SUP4*. However, when selection for growth in the absence of uracil was applied to cells containing this plasmid, little difference in Ura phenotype was seen in cells lacking Elongator function despite loss of *SUP4* efficiency in such cells (Huang *et al*., 2005), probably because selection for growth without uracil can drive up plasmid copy number. A 1225 bp EcoRI–NdeI fragment from pRS303-*URA3-SUP4* was therefore cloned between the same sites within the YIplac211 backbone (Gietz and Akio, 1988), followed by insertion of a 1245 bp EcoRI–SalI fragment carrying the *NatRMX* marker from pAG25 (Goldstein and McCusker, 1999) between the EcoRI and SalI sites. Finally, a synthetic DNA segment (Integrated DNA Technologies) was inserted between the unique HindIII and SalI sites of this new construct to generate pSB3. After linearizing pSB3 with MscI, the entire construct is flanked by *HIS3* sequences that allow targeting of a single copy to the wild-type *HIS3* locus or its *his3-11,15* and *his3Δ1* derivatives under ClonNat selection (see Fig. S1 in Supporting Information). pSB1 (in which the *URA3* gene lacks the *ochre* mutation) was generated similarly. In contrast to wild-type cells, Elongator-deficient cells containing integrated pSB3 showed strongly reduced ability to grow in the absence of added uracil, whereas no significant difference was observed using pSB1. Integration of pSB3 therefore confers the ability to read out the efficiency of *SUP4 ochre* suppression by monitoring growth in the absence of uracil.

### Fluorescence microscopy

For fixed cell imaging, overnight cultures were subcultured to log-phase (OD_600_ ∼ 1) at 30°C in the appropriate medium. Cells of each strain equivalent of 4.5 OD_600_ units were fixed by adding formaldehyde to 3.7% (v/v) for 30 min with gentle agitation. Cells were washed three times with 1× PBS and pipetted onto 0.2% (w/v) Concanavalin A (Sigma) treated coverslips. After immobilizing for 3–5 min, coverslips were placed on microscope slides with a small spot of antifade solution [0.5% (w/v) *p*-phenylenediamine, 20 mM Tris-HCl (pH 8.8), 90% (v/v) glycerol] and sealed with nail varnish. Cells were visualized on a Delta Vision RT microscope (Applied Precision) connected to a CCD camera (CoolsnapHQ / ICX285) using a 100× lens (Olympus 1X-71). 2D images (512 × 512) were captured with Bin 2 × 2 mode (0.132 μm pixel size) using an ET filter set (Chroma, 89006) to visualize GFP and mCherry signals. Flat field calibration was carried out before imaging for the GFP channel using a fluorescent calibration slide (488–519 nm, Applied Precision) and applied during image acquisition.

### Immunoprecipitation and Western blot analysis

Strains were grown to log phase (OD_600_ ∼ 1.0) and typically 50 OD_600_ units of cells were harvested by centrifugation, snap frozen using liquid nitrogen and then lysed by shaking vigorously at 4°C with 0.4 mm glass beads in cold Lysis Buffer [50 mM HEPES-KOH (pH 7.5), 2 mM MgCl_2_, 150 mM NaCl, 0.1% (v/v) Triton X-100, 50 mM NaF, 50 mM Na-βglycerophosphate] supplemented with Complete EDTA free Protease inhibitor (Roche), PhosSTOP (Roche), 1 mM AEBSF and 2 mM Na_3_VO_4_. All further steps were carried out at 4°C. Lysates were cleared by centrifugation at 14 000 rpm for 10 min and normalized using Bradford Coomassie reagent (Pierce). Protein extracts (∼ 0.5 ml) were pre-cleared with 25 μl Sepharose-4B beads (Sigma) for 30 min and then added to 50 μl Sepharose-4B beads conjugated to GFP–Trap (Chromotek) for 2 h with end-over rotation. GFP–Trap beads were washed three times with Lysis Buffer and proteins were eluted from the beads twice by adding 50 μl 2× Laemmli Sample Buffer and incubating at 75°C for 15 min, pooling the two eluates.

Protein samples prepared as above were analysed by Western blotting using anti–GFP (Roche, 11814460001), anti-HA (Santa-Cruz, sc-7392) and anti-Cdc28 (Santa-Cruz, sc-6709). Densitometry of bands was carried out using Aida Image Analyzer v.3.27 and Statistical analyses were carried out using SigmaPlot 12.0 (Systat Software, Inc.).

### Recombinant Elp1 expression and purification

The C-terminal domains of Elp1 and Elp1–KR9A including a C-terminal His_6_ tag were cloned into pGEX4T-1 and expressed in *E. coli* BL21 (DE3) (Stratagene). A plasmid encoding GST-His_6_ (a kind gift from Tom Owen-Hughes) was used to express GST-His_6_ for use as a control. Cultures were grown in Autoinduction Medium (Studier, 2005) at 30°C with shaking for 4–5 h and then the temperature was reduced to 25°C overnight. All further steps were carried out at 4°C. The cells were lysed in cold Lysis Buffer [20 mM Tris-HCl (pH 7.5), 300 mM NaCl] supplemented with Complete EDTA free Protease inhibitors (Roche) and cleared by centrifugation at 20 000 *g* for 30 min. Fusion proteins were purified using HisPur Cobalt resin (Thermo Scientific) and the eluate supplemented with 10 mM DTT before further purification with Glutathione-Sepharose Fast Flow 4 resin (GE Healthcare). Proteins were eluted from the glutathione resin using GST elution buffer [50 mM Tris-HCl (pH 8.0), 50 mM reduced glutathione] and diafiltered into Protein Binding Buffer [20 mM Tris-HCl (pH 7.5), 150 mM NaCl, 2 mM MgCl_2_].

### EMSA assays

Yeast tRNA (R9001, Sigma) was treated with Fast alkaline phosphatase (Fermentas) to remove the 5′ phosphate group before radiolabelling with [γ-^32^P]-ATP (Specific activity 6000 Ci mmol^−1^, Perkin Elmer) using T4 polynucleotide kinase (Fermentas). Unincorporated nucleotides were removed using a G-25 microspin column (GE healthcare). For EMSAs using radiolabelled tRNA, each binding reaction consisted of approximately 1 nM radiolabelled tRNA, mixed with the appropriate concentration of protein in Binding Buffer [20 mM Tris-HCl (pH 7.5), 150 mM NaCl, 2 mM MgCl_2,_ 8% (v/v) glycerol and 10 U Ribolock (Thermo scientific) RNase inhibitor] and allowed to equilibrate for 45 min at room temperature. Binding reactions were separated on 1.5% agarose gels in ultrapure 0.5× TBE Buffer (Life Technologies) at 100–120 V, cooling the gel apparatus in an ice bucket during electrophoresis. Gels were fixed and then dried at 50°C for 90 min on a vacuum gel drier (Bio-Rad) before exposure to Phosphoimager film. Bands were visualized by scanning using a fluorescent image analyser (FLA-5100 Fujifilm). Competitor EMSA experiments were performed similarly except that competitor was added to the binding reaction before the radiolabelled tRNA. Competitors used were ssDNA (5′-GCTGAAAGACTTGGTCTAAGC-3′) and poly(U) RNA (chain length 20). For estimation of dissociation constant from EMSA experiments (see Ryder *et al*., 2008), the conditions were as described except that approximately 0.1 nM [^32^P]-tRNA was used per binding reaction. Analysis of bands by densitometry was carried out using Aida Image Analyzer v.3.2.

To prepare yeast tRNA, total RNA was extracted from yeast using Tripure reagent (Roche) according to manufacturer's instructions and large RNAs (greater than 200 bp) were precipitated for 3 h at −20°C with 0.33 volume of 8 M LiCl. The small RNAs in the supernatant were precipitated overnight at −20°C with 0.1 volume of 3 M sodium acetate, 2.5 volumes of 100% ethanol, 20 μg glycogen and a final concentration of 10 mM MgCl_2_. The pellet was washed once in 75% ethanol before being air dried and dissolved in water. Small RNA preps were run on a 10% TBE-Urea gel (Novex) and the tRNA band was extracted and purified using a small-RNA PAGE recovery kit (Zymo Research).
