# Supplementary material for: A conserved and essential basic region mediates tRNA binding to the Elp1 subunit of the Saccharomyces cerevisiae Elongator complex
Source: Mol Microbiol. 2014 May 19;92(6):1227–42. doi: 10.1111/mmi.12624 (PMC4150532; doi:10.1111/mmi.12624)
Supplement: Supplementary file 1 — Additional supporting information may be found in the online version of this article at the publisher's web-site. [file mmi0092-1227-SD1.pdf]

Figure S1

A

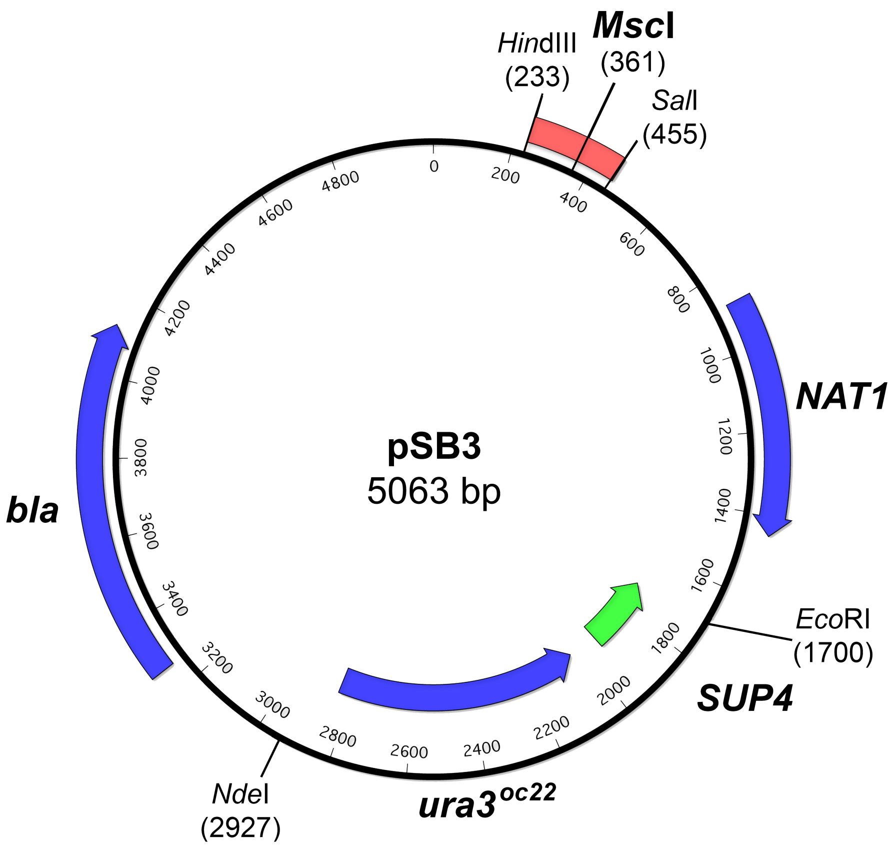

B

*Hind*III (495)

```
AAGCTTTGCAGAGGCTAGCAGAATTACCCTCCACGTTGATTGTCTGCGAGGCAAGAATGATCATCA
                                                           623  209
CCGTAGTGAGAGTGCGTTCAAGGCTCTTGCGGTTGCCATAAGAGAAGCCACCTCGCCCAATGG|CC
                                                             MscI
AAGCATTCCGGCTGGTCGCTAATCGTTGAGTGCATTGGTGACTTACACATAGACGACCATCACACC
ACTGAAGACTGCGGGATTGCTCTCGGTCgac
                         304 SalI
```

Figure S1. Construction of a plasmid for targeting single-copy integration of *SUP4* and a *ura3 ochre* allele to the *his3* locus. (A) Plasmid map showing the position of the restriction sites that define the different DNA sequences used in construction of the plasmid, together with the unique *Msc*I site used to target the construct in single copy to the *HIS3* locus. *bla*, ampicillin resistance; *NAT1*, ClonNat resistance (bracketed by *TEF* promoter and terminator sequences that are not shown); *ura3*^{oc22}, *ura3* allele in which codon 22 is substituted by an *ochre* termination codon; *SUP4*, tRNA^{Tyr}(UUA) ochre suppressor tRNA. (B) Sequence of the *Hind*III-*Sal*I fragment used for *HIS3* targetting. Upper-case letters correspond to the *HIS3* coding sequence positions indicated (numbered from the first base of the *HIS3* open reading frame), lower case letters represent additional bases required to generate the *Sal*I site. Linearization using *Msc*I (|, cut site) generates a fragment flanked by *HIS3* residues 209-304 and 495-623 that integrates in single copy at the *HIS3* locus.
